# Sero-Surveillance of Lyssavirus Specific Antibodies in Nigerian Fruit Bats (*Eidolon helvum*)

**DOI:** 10.3390/tropicalmed2030026

**Published:** 2017-07-09

**Authors:** Dinchi A. Tyem, Banenat B. Dogonyaro, Timothy A. Woma, Ernest Chuene Ngoepe, Claude Taurai Sabeta

**Affiliations:** 1 National Veterinary Research Institute, P.M.B. 1, Vom-Jos Plateau State, Nigeria; jasminetyem@yahoo.com (D.A.T.); bbdogonyaro@gmail.com (B.B.D); WomaT@up.ac.za (T.A.W.); 2 University of Pretoria, Faculty of Veterinary Sciences, Department of Veterinary Tropical Diseases, P Bag X04, Onderstepoort 0110, South Africa; 3 OIE Rabies Reference Laboratory, Agricultural Research Council-Onderstepoort Veterinary Research, Onderstepoort 0110, South Africa; NgoepeE@arc.agric.za

**Keywords:** lyssavirus, rabies, *Eidolon helvum*, Nigeria, blocking ELISA, phylogroup I, phylogroup II

## Abstract

The aetiological agent of rabies is a member of the *Lyssavirus* genus (*Rhabdoviridae* family, order *Mononegavirales*). The disease (rabies) is endemic in many parts of Asia and Africa and still remains an important public and veterinary health threat. In Nigeria, there is a dearth of information on the natural infection and/or exposure of bat species to lyssaviruses. Therefore, this study was undertaken to assess the prevalence of rabies virus (RABV) neutralizing antibodies in sera obtained from bats from the central Plateau and North-East Bauchi States in Nigeria. Two hundred serum samples were collected from Nigerian fruit bats from six different locations and tested for anti-RABV antibodies using a commercial blocking ELISA. Of the 200 bat serum samples collected, one batch consisting of 111 samples did not meet the validation criteria and hence was not included in the final analysis. Of the remaining 89, only three (3.4%) contained anti-lyssavirus antibodies, demonstrating a low prevalence of lyssavirus antibodies in the study population. In order to further understand the exposure of bat species to phylogroup II lyssaviruses (Lagos bat virus and Mokola virus), the same panel of samples will be tested for neutralizing antibodies to phylogroup II members, viruses that do not cross-neutralize with members of phylogroup I.

## 1. Introduction

Rabies is a fatal zoonotic disease and the aetiologic agent belongs to the genus *Lyssavirus* (*Rhabdoviridae* family) and order *Mononegavirales*. Despite rabies being a very old disease, it still commands great global veterinary and public health importance. There are presently fourteen recognised lyssavirus species in the genus and these include: rabies lyssavirus (RABV), Lagos bat lyssavirus (LBV), Mokola lyssavirus (MOKV), Duvenhage lyssavirus (DUVV), European bat 1 lyssavirus (EBLV-1), European bat 2 lyssavirus (EBLV-2), Australian bat lyssavirus (ABLV), Aravan lyssavirus (ARAV), Khujand lyssavirus (KHUV), Irkut lyssavirus (IRKV), West Caucasian bat lyssavirus (WCBV), Bokeloh bat lyssavirus (BBLV), Shimoni bat lyssavirus (SHIBV), and Ikoma lyssavirus (IKOV) [1,2]. Furthermore, two putative lyssavirus species, Lleida bat lyssavirus (LLEBV) [3] and Gannoruwa bat lyssavirus (GBLV) [4], have been identified in bat species and are still awaiting official classification. The viral species of this genus have been further sub-divided into three phylogroups based on their genetic distances, serologic cross-reactivity and/or pathogenicity studies in mouse model [5,6,7].

RABLV is found worldwide, and is responsible for the overwhelming majority of reported animal and human rabies cases [8,9,10]. Other lyssaviruses appear to have more restricted geographical and host range, with the majority having been isolated from bats with limited public and animal health implications. However, all lyssaviruses tested cause clinical disease indistinguishable from RABLV. In Nigeria, LBV was first isolated from the straw-coloured fruit bat *Eidolon helvum* in 1956 on Lagos Island [11]. A seroprevalence of 19% was demonstrated in this species (in Nigeria) by a different research group [12], whereas almost double that seroprevalence (37%) was observed in the same bat species in Ghana [13]. In contrast, other studies found an even higher seroprevalence, for instance in Kenya (40–67%), albeit from different bat colonies [14]. In Nigeria and throughout most of the African continent where bat populations are found, the natural infection of bat species with lyssaviruses is either unknown or poorly understood.

## 2. Materials and Methods

Ethical clearance (AEC/02/04/14) was granted by the Animal Ethical Committee (AEC) of the National Veterinary Research Institute, Vom, Nigeria, for the collection of blood samples from bats. Two hundred *E. helvum* were captured using mist nets from six different roosting sites in the central Plateau and North East Bauchi states of Nigeria with a view to assess the seroprevalence of RABV antibodies in this bat species. The bats were restrained manually and anesthetized with ketamine hydrochloride as described previously [15]. Blood samples were then collected (0.1–1 mL) through the jugular vein using 2 mL needle and syringe. The blood samples were then transferred into 5 mL sterile serum separator tubes via cold chain to the laboratory, where all the blood samples were centrifuged at 4000 rpm for 3 minutes, and thereafter serum samples were dispensed into sterile 2 mL screw capped cryovial tubes and stored at −80 °C until required.

This panel of serum samples was tested with an ELISA kit (BioPro rabies ELISA Ab kit, Prague, Czech Republic), a blocking ELISA for serological diagnostic of rabies lyssavirus antibody in serum or plasma of domesticated and wild animals [16]. In brief, the serum samples and controls (rabies positive control serum, rabies control serum 1, rabies control serum 2, rabies control serum 1 and rabies negative control) were diluted two-fold using sample diluent buffer provided in the kit. At least 100 µL of each dilution were distributed into the respective well of the plate. Thereafter, the plate was sealed with plate sealer and incubated at 4–8 °C overnight (O/N) with gentle shaking on orbital shaker. The plate was washed six times with washing solution using automated washer (BioTek, Winooski, VT, USA) and excess buffer was absorbed on paper towel. The biotinylated anti-rabies antibody was diluted to a working dilution of 1:100 and 100 µL was distributed into each well. The plate was covered with a plate sealer and incubated at 37 ± 2 °C for 30 minutes with gentle shaking as described before, then washed four times. Excess buffer was removed by tapping on paper towel. The streptavidin peroxidase conjugate was diluted 100-fold and 100 µL was dispensed into each well. The plate was sealed and incubated as previously. The plate was washed four times with washing solution and excess buffer was removed as before. At least 100 µL of ready-to-use TMB substrate was added to each well and the plate was incubated at room temperature for 15–30 min, with gently shaking on an orbital shaker away from direct sunlight. Thereafter, the reaction was stopped with 50 µL of stop solution per well and the results were read at 450 nm using an ELISA reader. The optical density (OD) values were expressed as percentage blocking by using the following formula:

PB% = [OD NC − OD SAMPLE/OD NC − OD PC] × 100
(1)

The results were accepted only when the OD of a negative control serum of higher than 1 was obtained and the difference between the means of OD of negative and positive control serum samples was equal or higher than 0.8.

## 3. Results and Discussion

**Figure 1:**
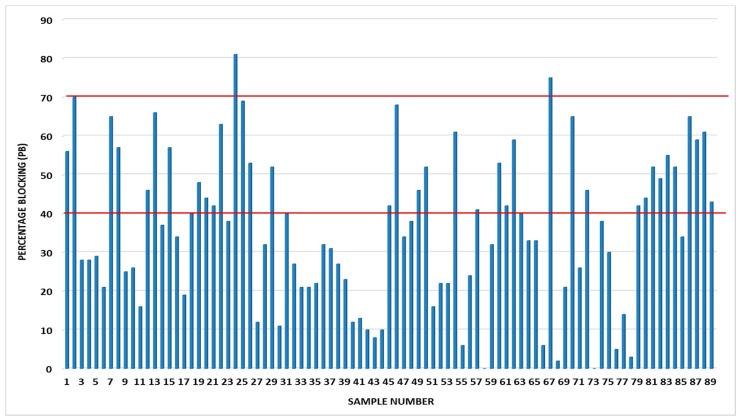
The distribution of percentage blocking values obtained from some of the bat sera obtained from *Eidolon helvum* from the central Plateau and North East Bauchi States of Nigeria. The red bars are the cut-off values (40% and 70%) with the former considered positive for rabies antibodies, and 70% considered as serum sample with antibody level equal to or higher than 0.5 IU/mL based on the FAVN test.

Using the set cut-off values (of 40% and 70%) for this ELISA antibody kit, with the latter corresponding to the gold standard method fluorescent antibody virus neutralization test (FAVNT) cut-off value of 0.5 IU/mL, it could be shown that only two serum samples were on the borderline and an equal number just above the cut-off value (Figure 1). Of the 200 serum samples analysed, only 89 met the validation criteria specified by the manufacturer and included for analysis. Three serum samples (sample numbers 2, 24, and 67), i.e., 3.4% of the study sample, contained lyssavirus-specific antibodies with percentage blockings (PBs) of 70%, 81% and 75% respectively (Figure 1). 

The three serum samples were subsequently shown to contain RABV-neutralising antibodies just below the 0.5 IU/mL cut-off using a laboratory strain of rabies virus (challenge virus standard). The 70% cut-off value demonstrates adequate seroconversion for international movement of pets using a fluorescent antibody neutralisation test [17]. In order to further understand the exposure of bat species to phylogroup II lyssaviruses (Lagos bat virus and Mokola virus), the same panel of samples (particularly those above 40%) can be tested for neutralizing antibodies to phylogroup II members. Data from a study carried out in Ibadan (western Nigeria) in 1990 showed the presence of RABV neutralizing antibodies in the sera of fruit bats [18]. However, given *Eidolon helvum* has been associated with a reservoir of LBV, it is crucial that the panel of samples be tested against this (LBV) lyssavirus [12,13]. These data therefore indicated possible cross-reactivity of antibodies amongst specific phylogroups within the *Lyssavirus* genus.
